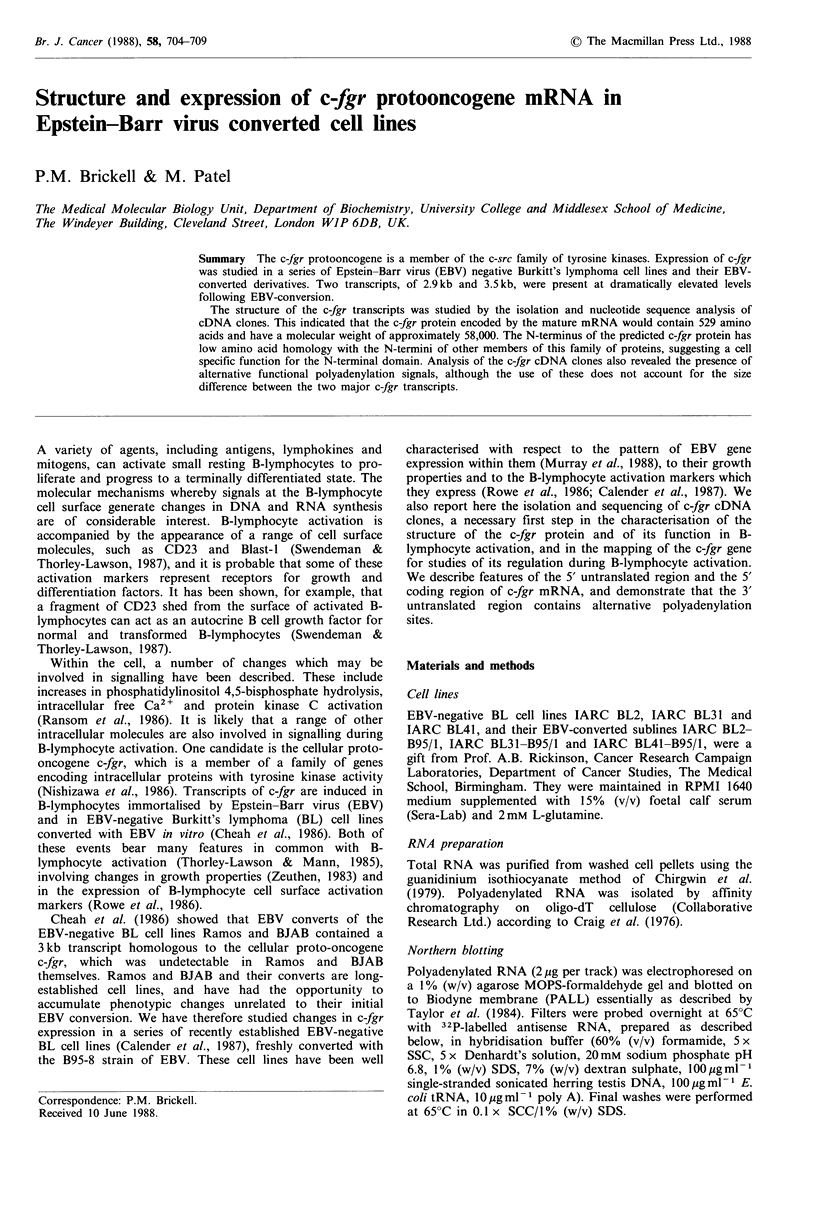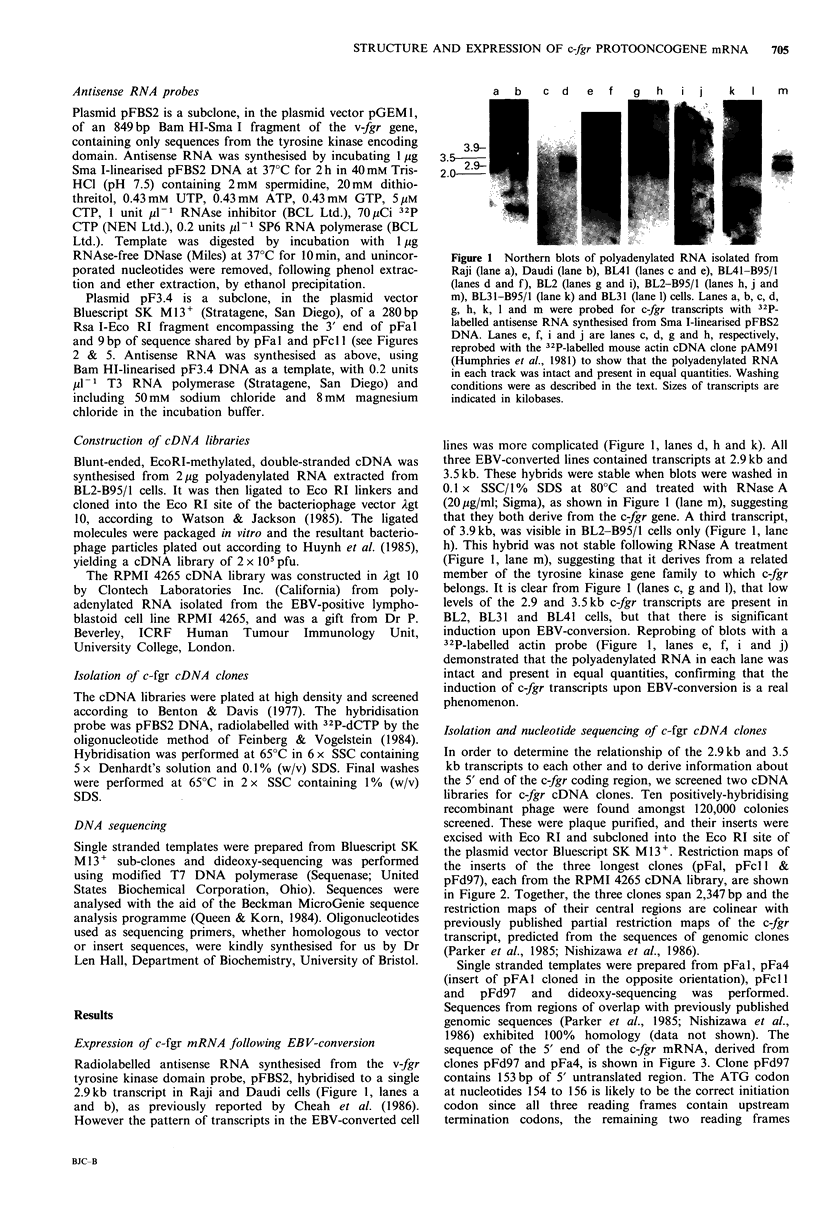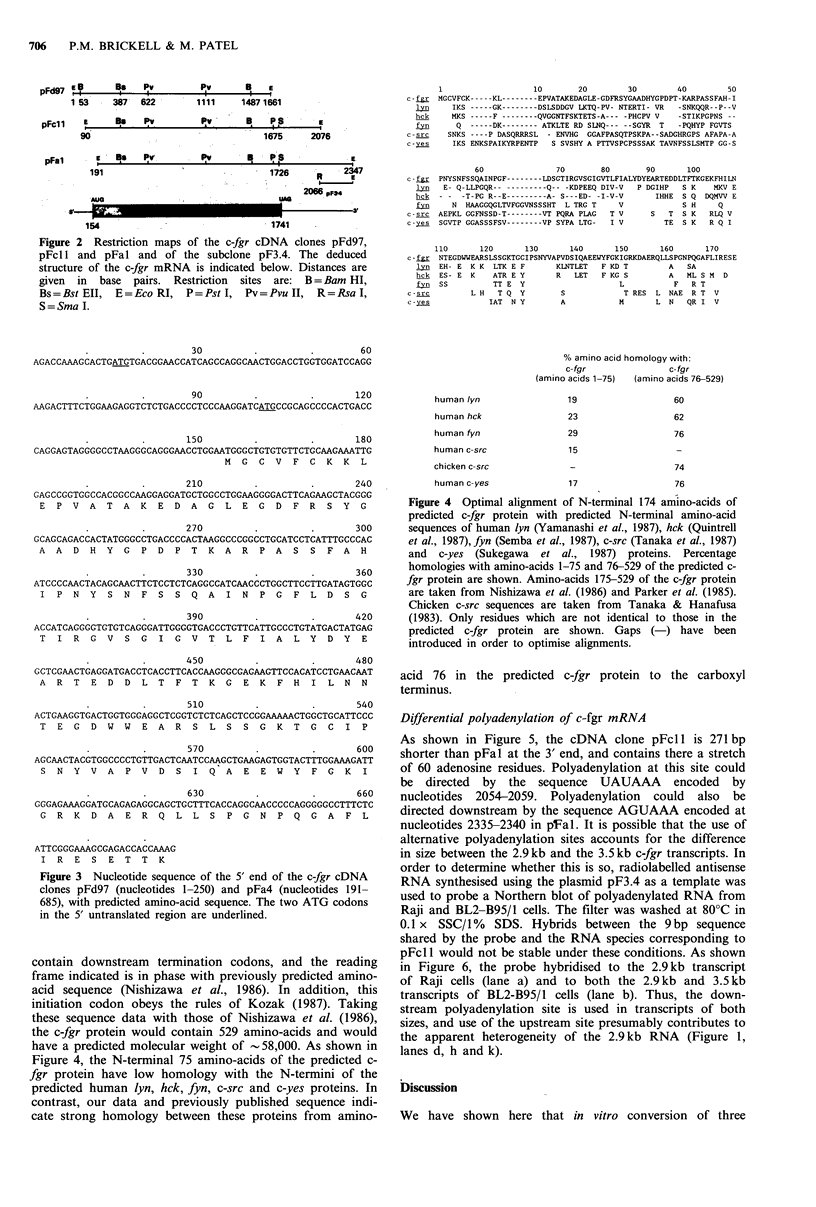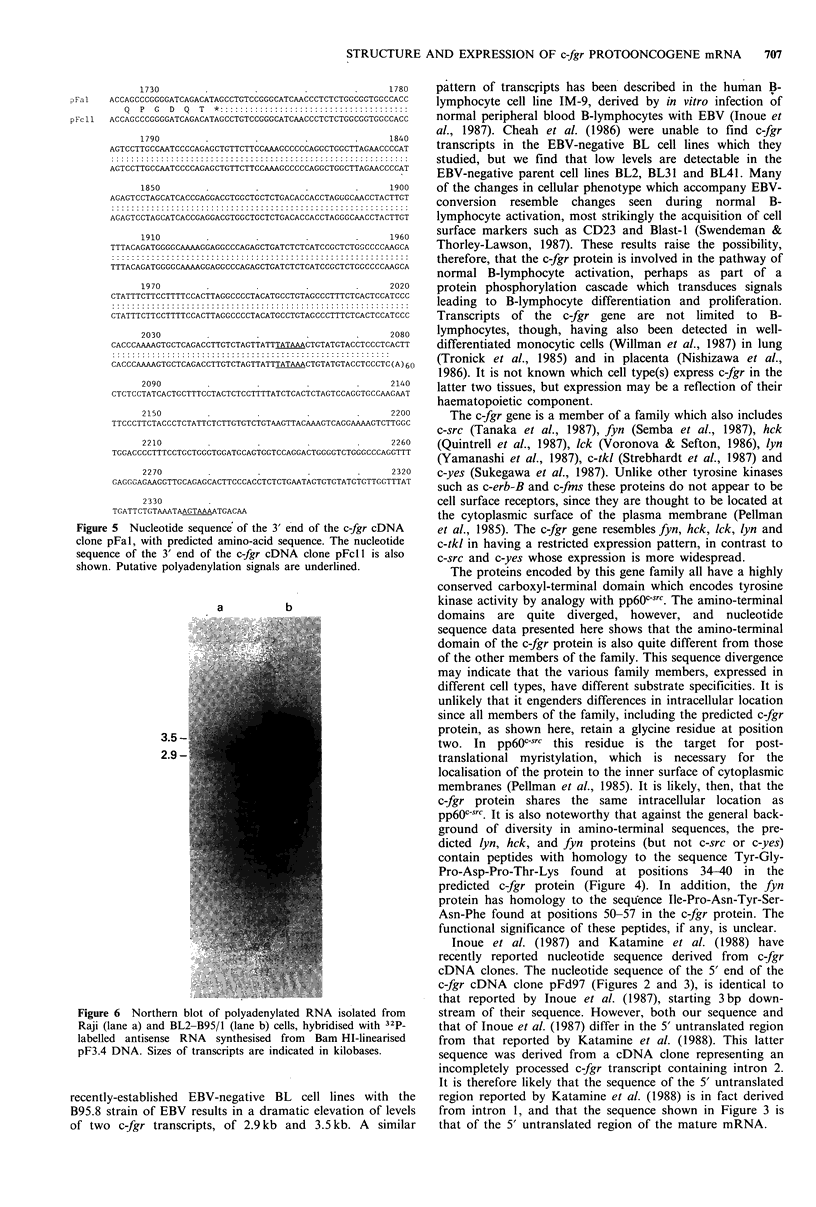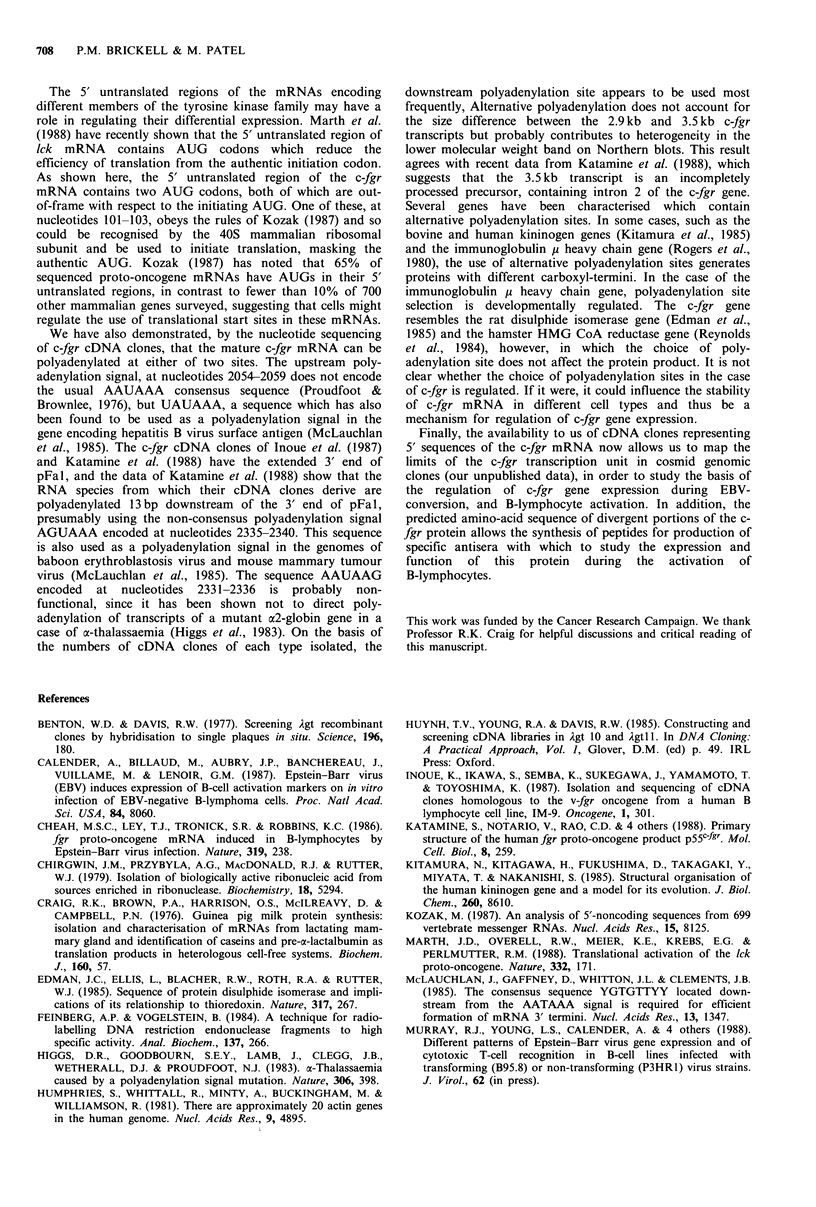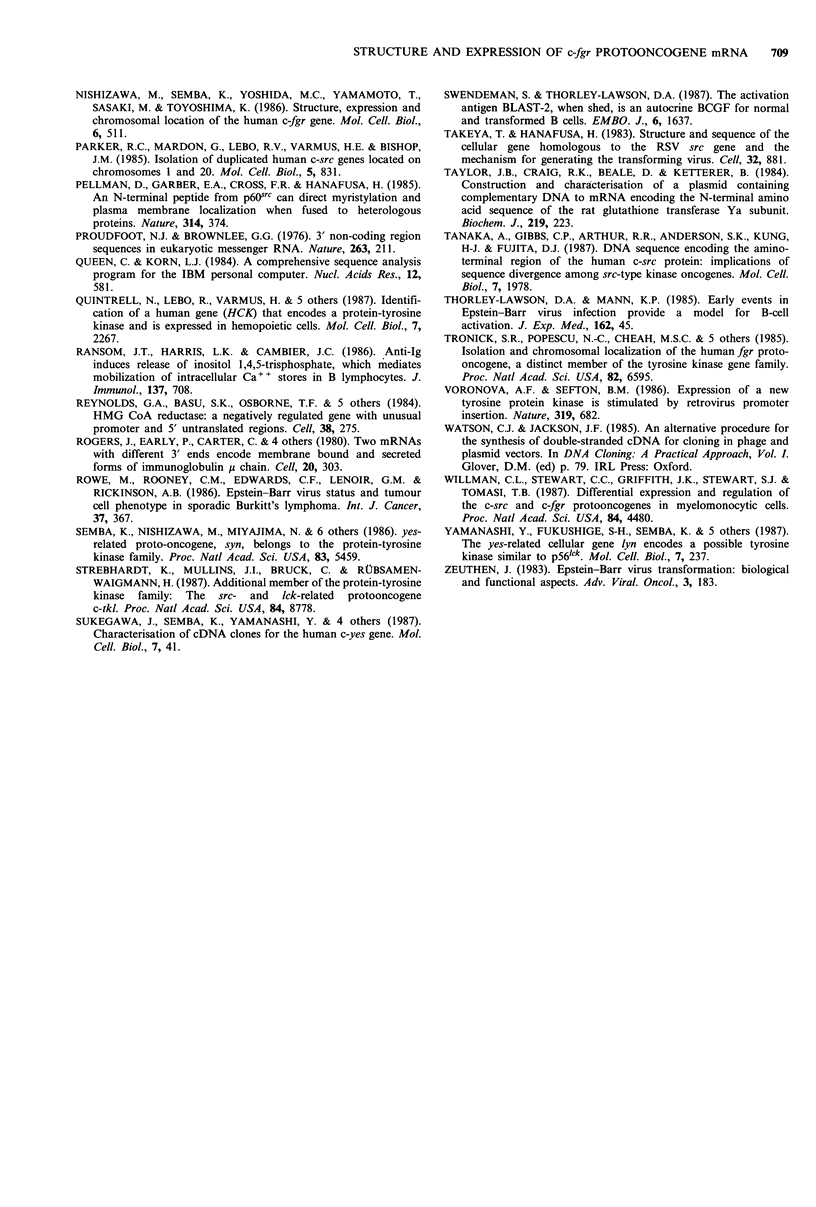# Structure and expression of c-fgr protooncogene mRNA in Epstein-Barr virus converted cell lines.

**DOI:** 10.1038/bjc.1988.294

**Published:** 1988-12

**Authors:** P. M. Brickell, M. Patel

**Affiliations:** Department of Biochemistry, University College and Middlesex School of Medicine, London, UK.

## Abstract

**Images:**


### Structure and expression of c-*fgr* protooncogene mRNA in Epstein–Barr virus converted cell lines

P.M. Brickell & M. Patel

*The Medical Molecular Biology Unit, Department of Biochemistry, University College and Middlesex School of Medicine, The Windeyer Building, Cleveland Street, London W1P 6DB, UK.*

**Summary** The c-*fgr* protooncogene is a member of the c-*src* family of tyrosine kinases. Expression of c-*fgr* was studied in a series of Epstein–Barr virus (EBV) negative Burkitt's lymphoma cell lines and their EBV-converted derivatives. Two transcripts, of 2.9 kb and 3.5 kb, were present at dramatically elevated levels following EBV-conversion.

The structure of the c-*fgr* transcripts was studied by the isolation and nucleotide sequence analysis of cDNA clones. This indicated that the c-*fgr* protein encoded by the mature mRNA would contain 529 amino acids and have a molecular weight of approximately 58,000. The N-terminus of the predicted c-*fgr* protein has low amino acid homology with the N-termini of other members of this family of proteins, suggesting a cell specific function for the N-terminal domain. Analysis of the c-*fgr* cDNA clones also revealed the presence of alternative functional polyadenylation signals, although the use of these does not account for the size difference between the two major c-*fgr* transcripts.

A variety of agents, including antigens, lymphokines and mitogens, can activate small resting B-lymphocytes to proliferate and progress to a terminally differentiated state. The molecular mechanisms whereby signals at the B-lymphocyte cell surface generate changes in DNA and RNA synthesis are of considerable interest. B-lymphocyte activation is accompanied by the appearance of a range of cell surface molecules, such as CD23 and Blast-1 (Swendeman & Thorley-Lawson, 1987), and it is probable that some of these activation markers represent receptors for growth and differentiation factors. It has been shown, for example, that a fragment of CD23 shed from the surface of activated B-lymphocytes can act as an autocrine B cell growth factor for normal and transformed B-lymphocytes (Swendeman & Thorley-Lawson, 1987).

Within the cell, a number of changes which may be involved in signalling have been described. These include increases in phosphatidylinositol 4,5-bisphosphate hydrolysis, intracellular free $Ca^{2+}$ and protein kinase C activation (Ransom *et al.*, 1986). It is likely that a range of other intracellular molecules are also involved in signalling during B-lymphocyte activation. One candidate is the cellular proto-oncogene c-*fgr*, which is a member of a family of genes encoding intracellular proteins with tyrosine kinase activity (Nishizawa *et al.*, 1986). Transcripts of c-*fgr* are induced in B-lymphocytes immortalised by Epstein–Barr virus (EBV) and in EBV-negative Burkitt's lymphoma (BL) cell lines converted with EBV *in vitro* (Cheah *et al.*, 1986). Both of these events bear many features in common with B-lymphocyte activation (Thorley-Lawson & Mann, 1985), involving changes in growth properties (Zeuthen, 1983) and in the expression of B-lymphocyte cell surface activation markers (Rowe *et al.*, 1986).

Cheah *et al.* (1986) showed that EBV converts of the EBV-negative BL cell lines Ramos and BJAB contained a 3 kb transcript homologous to the cellular proto-oncogene c-*fgr*, which was undetectable in Ramos and BJAB themselves. Ramos and BJAB and their converts are long-established cell lines, and have had the opportunity to accumulate phenotypic changes unrelated to their initial EBV conversion. We have therefore studied changes in c-*fgr* expression in a series of recently established EBV-negative BL cell lines (Calender *et al.*, 1987), freshly converted with the B95-8 strain of EBV. These cell lines have been well characterised with respect to the pattern of EBV gene expression within them (Murray *et al.*, 1988), to their growth properties and to the B-lymphocyte activation markers which they express (Rowe *et al.*, 1986; Calender *et al.*, 1987). We also report here the isolation and sequencing of c-*fgr* cDNA clones, a necessary first step in the characterisation of the structure of the c-*fgr* protein and of its function in B-lymphocyte activation, and in the mapping of the c-*fgr* gene for studies of its regulation during B-lymphocyte activation. We describe features of the 5′ untranslated region and the 5′ coding region of c-*fgr* mRNA, and demonstrate that the 3′ untranslated region contains alternative polyadenylation sites.

Correspondence: P.M. Brickell.
Received 10 June 1988.

### Materials and methods

### *Cell lines*

EBV-negative BL cell lines IARC BL2, IARC BL31 and IARC BL41, and their EBV-converted sublines IARC BL2–B95/1, IARC BL31–B95/1 and IARC BL41–B95/1, were a gift from Prof. A.B. Rickinson, Cancer Research Campaign Laboratories, Department of Cancer Studies, The Medical School, Birmingham. They were maintained in RPMI 1640 medium supplemented with 15% (v/v) foetal calf serum (Sera-Lab) and 2 mM L-glutamine.

### *RNA preparation*

Total RNA was purified from washed cell pellets using the guanidinium isothiocyanate method of Chirgwin *et al.* (1979). Polyadenylated RNA was isolated by affinity chromatography on oligo-dT cellulose (Collaborative Research Ltd.) according to Craig *et al.* (1976).

### *Northern blotting*

Polyadenylated RNA (2 μg per track) was electrophoresed on a 1% (w/v) agarose MOPS-formaldehyde gel and blotted on to Biodyne membrane (PALL) essentially as described by Taylor *et al.* (1984). Filters were probed overnight at 65°C with $^{32}$P-labelled antisense RNA, prepared as described below, in hybridisation buffer (60% (v/v) formamide, 5× SSC, 5× Denhardt's solution, 20 mM sodium phosphate pH 6.8, 1% (w/v) SDS, 7% (w/v) dextran sulphate, 100 μg ml$^{-1}$ single-stranded sonicated herring testis DNA, 100 μg ml$^{-1}$ *E. coli* tRNA, 10 μg ml$^{-1}$ poly A). Final washes were performed at 65°C in 0.1× SCC/1% (w/v) SDS.

*Antisense RNA probes*

Plasmid pFBS2 is a subclone, in the plasmid vector pGEM1, of an 849 bp Bam HI-Sma I fragment of the v-*fgr* gene, containing only sequences from the tyrosine kinase encoding domain. Antisense RNA was synthesised by incubating 1 $\mu$g Sma I-linearised pFBS2 DNA at 37°C for 2 h in 40 mM Tris-HCl (pH 7.5) containing 2 mM spermidine, 20 mM dithiothreitol, 0.43 mM UTP, 0.43 mM ATP, 0.43 mM GTP, 5 $\mu$M CTP, 1 unit $\mu l^{-1}$ RNAse inhibitor (BCL Ltd.), 70 $\mu$Ci $^{32}$P CTP (NEN Ltd.), 0.2 units $\mu l^{-1}$ SP6 RNA polymerase (BCL Ltd.). Template was digested by incubation with 1 $\mu$g RNAse-free DNase (Miles) at 37°C for 10 min, and unincorporated nucleotides were removed, following phenol extraction and ether extraction, by ethanol precipitation.

Plasmid pF3.4 is a subclone, in the plasmid vector Bluescript SK M13$^+$ (Stratagene, San Diego), of a 280 bp Rsa I-Eco RI fragment encompassing the 3′ end of pFa1 and 9 bp of sequence shared by pFa1 and pFc11 (see Figures 2 & 5. Antisense RNA was synthesised as above, using Bam HI-linearised pF3.4 DNA as a template, with 0.2 units $\mu l^{-1}$ T3 RNA polymerase (Stratagene, San Diego) and including 50 mM sodium chloride and 8 mM magnesium chloride in the incubation buffer.

*Construction of cDNA libraries*

Blunt-ended, EcoRI-methylated, double-stranded cDNA was synthesised from 2 $\mu$g polyadenylated RNA extracted from BL2-B95/1 cells. It was then ligated to Eco RI linkers and cloned into the Eco RI site of the bacteriophage vector $\lambda$gt 10, according to Watson & Jackson (1985). The ligated molecules were packaged *in vitro* and the resultant bacteriophage particles plated out according to Huynh *et al.* (1985), yielding a cDNA library of $2 \times 10^5$ pfu.

The RPMI 4265 cDNA library was constructed in $\lambda$gt 10 by Clontech Laboratories Inc. (California) from polyadenylated RNA isolated from the EBV-positive lymphoblastoid cell line RPMI 4265, and was a gift from Dr P. Beverley, ICRF Human Tumour Immunology Unit, University College, London.

*Isolation of* c-fgr *cDNA clones*

The cDNA libraries were plated at high density and screened according to Benton & Davis (1977). The hybridisation probe was pFBS2 DNA, radiolabelled with $^{32}$P-dCTP by the oligonucleotide method of Feinberg & Vogelstein (1984). Hybridisation was performed at 65°C in 6 × SSC containing 5 × Denhardt's solution and 0.1% (w/v) SDS. Final washes were performed at 65°C in 2 × SSC containing 1% (w/v) SDS.

*DNA sequencing*

Single stranded templates were prepared from Bluescript SK M13$^+$ sub-clones and dideoxy-sequencing was performed using modified T7 DNA polymerase (Sequenase; United States Biochemical Corporation, Ohio). Sequences were analysed with the aid of the Beckman MicroGenie sequence analysis programme (Queen & Korn, 1984). Oligonucleotides used as sequencing primers, whether homologous to vector or insert sequences, were kindly synthesised for us by Dr Len Hall, Department of Biochemistry, University of Bristol.

### Results

*Expression of* c-fgr *mRNA following EBV-conversion*

Radiolabelled antisense RNA synthesised from the v-*fgr* tyrosine kinase domain probe, pFBS2, hybridised to a single 2.9 kb transcript in Raji and Daudi cells (Figure 1, lanes a and b), as previously reported by Cheah *et al.* (1986). However the pattern of transcripts in the EBV-converted cell lines was more complicated (Figure 1, lanes d, h and k). All three EBV-converted lines contained transcripts at 2.9 kb and 3.5 kb. These hybrids were stable when blots were washed in 0.1 × SSC/1% SDS at 80°C and treated with RNase A (20 $\mu$g/ml; Sigma), as shown in Figure 1 (lane m), suggesting that they both derive from the c-*fgr* gene. A third transcript, of 3.9 kb, was visible in BL2–B95/1 cells only (Figure 1, lane h). This hybrid was not stable following RNase A treatment (Figure 1, lane m), suggesting that it derives from a related member of the tyrosine kinase gene family to which c-*fgr* belongs. It is clear from Figure 1 (lanes c, g and l), that low levels of the 2.9 and 3.5 kb c-*fgr* transcripts are present in BL2, BL31 and BL41 cells, but that there is significant induction upon EBV-conversion. Reprobing of blots with a $^{32}$P-labelled actin probe (Figure 1, lanes e, f, i and j) demonstrated that the polyadenylated RNA in each lane was intact and present in equal quantities, confirming that the induction of c-*fgr* transcripts upon EBV-conversion is a real phenomenon.

**Figure 1** Northern blots of polyadenylated RNA isolated from Raji (lane a), Daudi (lane b), BL41 (lanes c and e), BL41–B95/1 (lanes d and f), BL2 (lanes g and i), BL2–B95/1 (lanes h, j and m), BL31–B95/1 (lane k) and BL31 (lane l) cells. Lanes a, b, c, d, g, h, k, l and m were probed for c-*fgr* transcripts with $^{32}$P-labelled antisense RNA synthesised from Sma I-linearised pFBS2 DNA. Lanes e, f, i and j are lanes c, d, g and h, respectively, reprobed with the $^{32}$P-labelled mouse actin cDNA clone pAM91 (Humphries *et al.*, 1981) to show that the polyadenylated RNA in each track was intact and present in equal quantities. Washing conditions were as described in the text. Sizes of transcripts are indicated in kilobases.

*Isolation and nucleotide sequencing of* c-fgr *cDNA clones*

In order to determine the relationship of the 2.9 kb and 3.5 kb transcripts to each other and to derive information about the 5′ end of the c-*fgr* coding region, we screened two cDNA libraries for c-*fgr* cDNA clones. Ten positively-hybridising recombinant phage were found amongst 120,000 colonies screened. These were plaque purified, and their inserts were excised with Eco RI and subcloned into the Eco RI site of the plasmid vector Bluescript SK M13$^+$. Restriction maps of the inserts of the three longest clones (pFa1, pFc11 & pFd97), each from the RPMI 4265 cDNA library, are shown in Figure 2. Together, the three clones span 2,347 bp and the restriction maps of their central regions are colinear with previously published partial restriction maps of the c-*fgr* transcript, predicted from the sequences of genomic clones (Parker *et al.*, 1985; Nishizawa *et al.*, 1986).

Single stranded templates were prepared from pFa1, pFa4 (insert of pFA1 cloned in the opposite orientation), pFc11 and pFd97 and dideoxy-sequencing was performed. Sequences from regions of overlap with previously published genomic sequences (Parker *et al.*, 1985; Nishizawa *et al.*, 1986) exhibited 100% homology (data not shown). The sequence of the 5′ end of the c-*fgr* mRNA, derived from clones pFd97 and pFa4, is shown in Figure 3. Clone pFd97 contains 153 bp of 5′ untranslated region. The ATG codon at nucleotides 154 to 156 is likely to be the correct initiation codon since all three reading frames contain upstream termination codons, the remaining two reading frames

**Figure 2** Restriction maps of the c-*fgr* cDNA clones pFd97, pFc11 and pFa1 and of the subclone pF3.4. The deduced structure of the c-*fgr* mRNA is indicated below. Distances are given in base pairs. Restriction sites are: B = *Bam* HI, Bs = *Bst* EII, E = *Eco* RI, P = *Pst* I, Pv = *Pvu* II, R = *Rsa* I, S = *Sma* I.

```
          .         .        30         .         .        60
AGACCAAAGCACTGATGTGACGGAACCATCAGCCAGGCAACTGGACCTGGTGGATCCAGG

          .         .        90         .         .       120
AAGACTTTCTGGAAGAGGTCTCTGACCCCTCCCAAGGATCATGCCGCAGCCCCACTGACC

          .         .       150         .         .       180
CAGGAGTAGGGGCCTAAGGGCAGGGAACCTGGAATGGGCTGTGTGTTCTGCAAGAAATTG
                                     M  G  C  V  F  C  K  K  L

          .         .       210         .         .       240
GAGCCGGTGGCCACGGCCAAGGAGGATGCTGGCCTGGAAGGGGACTTCAGAAGCTACGGG
 E  P  V  A  T  A  K  E  D  A  G  L  E  G  D  F  R  S  Y  G

          .         .       270         .         .       300
GCAGCAGACCACTATGGGCCTGACCCCACTAAGGCCCGGCCTGCATCCTCATTTGCCCAC
 A  A  D  H  Y  G  P  D  P  T  K  A  R  P  A  S  S  F  A  H

          .         .       330         .         .       360
ATCCCCAACTACAGCAACTTCTCCTCTCAGGCCATCAACCCTGGCTTCCTTGATAGTGGC
 I  P  N  Y  S  N  F  S  S  Q  A  I  N  P  G  F  L  D  S  G

          .         .       390         .         .       420
ACCATCAGGGGTGTGTCAGGGATTGGGGTGACCCTGTTCATTGCCCTGTATGACTATGAG
 T  I  R  G  V  S  G  I  G  V  T  L  F  I  A  L  Y  D  Y  E

          .         .       450         .         .       480
GCTCGAACTGAGGATGACCTCACCTTCACCAAGGGCGAGAAGTTCCACATCCTGAACAAT
 A  R  T  E  D  D  L  T  F  T  K  G  E  K  F  H  I  L  N  N

          .         .       510         .         .       540
ACTGAAGGTGACTGGTGGGAGGCTCGGTCTCTCAGCTCCGGAAAAACTGGCTGCATTCCC
 T  E  G  D  W  W  E  A  R  S  L  S  S  G  K  T  G  C  I  P

          .         .       570         .         .       600
AGCAACTACGTGGCCCCTGTTGACTCAATCCAAGCTGAAGAGTGGTACTTTGGAAAGATT
 S  N  Y  V  A  P  V  D  S  I  Q  A  E  E  W  Y  F  G  K  I

          .         .       630         .         .       660
GGGAGAAAGGATGCAGAGAGGCAGCTGCTTTCACCAGGCAACCCCCAGGGGGCCTTTCTC
 G  R  K  D  A  E  R  Q  L  L  S  P  G  N  P  Q  G  A  F  L

          .         .
ATTCGGGAAAGCGAGACCACCAAAG
 I  R  E  S  E  T  T  K
```

**Figure 3** Nucleotide sequence of the 5′ end of the c-*fgr* cDNA clones pFd97 (nucleotides 1–250) and pFa4 (nucleotides 191–685), with predicted amino-acid sequence. The two ATG codons in the 5′ untranslated region are underlined.

contain downstream termination codons, and the reading frame indicated is in phase with previously predicted amino-acid sequence (Nishizawa *et al.*, 1986). In addition, this initiation codon obeys the rules of Kozak (1987). Taking these sequence data with those of Nishizawa *et al.* (1986), the c-*fgr* protein would contain 529 amino-acids and would have a predicted molecular weight of ~58,000. As shown in Figure 4, the N-terminal 75 amino-acids of the predicted c-*fgr* protein have low homology with the N-termini of the predicted human *lyn*, *hck*, *fyn*, c-*src* and c-*yes* proteins. In contrast, our data and previously published sequence indicate strong homology between these proteins from amino-

```
       1                10        20        30        40        50
c-fgr  MGCVFCK-----KL--------EPVATAKEDAGLE-GDFRSYGAADHYGPDPT-KARPASSFAH-I
  lyn     IKS -----GK--------DSLSDDGV LKTQ-PV- NTERTI- VR   -SNKQQR--P--V
  hck     MKS -----F --------QVGGNTFSKTETS-A--- -PHCPV V    -STIKPGPNS --
  fyn      Q  -----DK-------- ATKLTE RD SLNQ--- --SGYR  T   -PQHYP FGVTS
c-src    SNKS ----P DASQRRRSL  - ENVHG  GGAFPASQTPSKPA--SADGHRGPS AFAPA-A
c-yes     IKS ENKSPAIKYRPENTP   S SVSHY A PTTVSPCPSSSAK TAVNFSSLSMTP GG-S

                60                70        80        90       100
c-fgr  PNYSNFSSQAINPGF--------LDSGTIRGVSGIGVTLFIALYDYEARTEDDLTFTKGEKFHILN
  lyn   E- Q-LLPGQR-- ---------Q-- -KDPEEQ DIV-V   P DGIHP   S K    MKV E
  hck  - -  -T-PG R--E---------A- S---ED- -I-V-V       IHHE  S Q  DQMVV E
  fyn     N  HAAGGQGLTVFGGVNSSSHT  L TRG T     V             S H     Q
c-src  AEPKL GGFNSSD-T--------VT PQRA PLAG   T V      S   T  S K   RLQ V
c-yes  SGVTP GGASSSFSV--------VP SYPA LTG-   I V          TE  S K   R Q I

      110       120       130       140       150       160       170
c-fgr  NTEGDWWEARSLSSGKTGCIPSNYVAPVDSIQAEEWYFGKIGRKDAERQLLSPGNPQGAFLIRESE
  lyn  EH-  E  K K  LTK E F       KLNTLET   F KD T         A   SA
  hck  ES-  E  K    ATR E Y       R   LET   F KG S         A   ML S M  D
  fyn  SS           TT E  Y                    L           F   R T
c-src          L H   T Q  Y        S              T RES  L  NAE  R T  V
c-yes                IAT  N Y        A               M        L  N   QR I  V
```

| | % amino acid homology with: c-*fgr* (amino acids 1–75) | c-*fgr* (amino acids 76–529) |
|---|---|---|
| human *lyn* | 19 | 60 |
| human *hck* | 23 | 62 |
| human *fyn* | 29 | 76 |
| human c-*src* | 15 | – |
| chicken c-*src* | – | 74 |
| human c-*yes* | 17 | 76 |

**Figure 4** Optimal alignment of N-terminal 174 amino-acids of predicted c-*fgr* protein with predicted N-terminal amino-acid sequences of human *lyn* (Yamanashi *et al.*, 1987), *hck* (Quintrell *et al.*, 1987), *fyn* (Semba *et al.*, 1987), c-*src* (Tanaka *et al.*, 1987) and c-*yes* (Sukegawa *et al.*, 1987) proteins. Percentage homologies with amino-acids 1–75 and 76–529 of the predicted c-*fgr* protein are shown. Amino-acids 175–529 of the c-*fgr* protein are taken from Nishizawa *et al.* (1986) and Parker *et al.* (1985). Chicken c-*src* sequences are taken from Tanaka & Hanafusa (1983). Only residues which are not identical to those in the predicted c-*fgr* protein are shown. Gaps (—) have been introduced in order to optimise alignments.

acid 76 in the predicted c-*fgr* protein to the carboxyl terminus.

### *Differential polyadenylation of c-*fgr *mRNA*

As shown in Figure 5, the cDNA clone pFc11 is 271 bp shorter than pFa1 at the 3′ end, and contains there a stretch of 60 adenosine residues. Polyadenylation at this site could be directed by the sequence UAUAAA encoded by nucleotides 2054–2059. Polyadenylation could also be directed downstream by the sequence AGUAAA encoded at nucleotides 2335–2340 in pFa1. It is possible that the use of alternative polyadenylation sites accounts for the difference in size between the 2.9 kb and the 3.5 kb c-*fgr* transcripts. In order to determine whether this is so, radiolabelled antisense RNA synthesised using the plasmid pF3.4 as a template was used to probe a Northern blot of polyadenylated RNA from Raji and BL2-B95/1 cells. The filter was washed at 80°C in 0.1 × SSC/1% SDS. Hybrids between the 9 bp sequence shared by the probe and the RNA species corresponding to pFc11 would not be stable under these conditions. As shown in Figure 6, the probe hybridised to the 2.9 kb transcript of Raji cells (lane a) and to both the 2.9 kb and 3.5 kb transcripts of BL2-B95/1 cells (lane b). Thus, the downstream polyadenylation site is used in transcripts of both sizes, and use of the upstream site presumably contributes to the apparent heterogeneity of the 2.9 kb RNA (Figure 1, lanes d, h and k).

### Discussion

We have shown here that *in vitro* conversion of three

```
            1730          .          .          .          .       1780
pFal    ACCAGCCCGGGGATCAGACATAGCCTGTCCGGGCATCAACCCTCTCTGGCGGTGGCCACC
          Q  P  G  D  Q  T  *:::::::::::::::::::::::::::::::::::::::
pFcll   ACCAGCCCGGGGATCAGACATAGCCTGTCCGGGCATCAACCCTCTCTGGCGGTGGCCACC

            1790          .          .          .          .       1840
        AGTCCTTGCCAATCCCCAGAGCTGTTCTTCCAAAGCCCCCAGGCTGGCTTAGAACCCCAT
        ::::::::::::::::::::::::::::::::::::::::::::::::::::::::::::
        AGTCCTTGCCAATCCCCAGAGCTGTTCTTCCAAAGCCCCCAGGCTGGCTTAGAACCCCAT

            1850          .          .          .          .       1900
        AGAGTCCTAGCATCACCGAGGACGTGGCTGCTCTGACACCACCTAGGGCAACCTACTTGT
        ::::::::::::::::::::::::::::::::::::::::::::::::::::::::::::
        AGAGTCCTAGCATCACCGAGGACGTGGCTGCTCTGACACCACCTAGGGCAACCTACTTGT

            1910          .          .          .          .       1960
        TTTACAGATGGGGCAAAAGGAGGCCCAGAGCTGATCTCTCATCCGCTCTGGCCCCAAGCA
        ::::::::::::::::::::::::::::::::::::::::::::::::::::::::::::
        TTTACAGATGGGGCAAAAGGAGGCCCAGAGCTGATCTCTCATCCGCTCTGGCCCCAAGCA

            1970          .          .          .          .       2020
        CTATTTCTTCCTTTTCCACTTAGGCCCCTACATGCCTGTAGCCCTTTCTCACTCCATCCC
        ::::::::::::::::::::::::::::::::::::::::::::::::::::::::::::
        CTATTTCTTCCTTTTCCACTTAGGCCCCTACATGCCTGTAGCCCTTTCTCACTCCATCCC

            2030          .          .          .          .       2080
        CACCCAAAAGTGCTCAGACCTTGTCTAGTTATTTATAAACTGTATGTACCTCCCTCACTT
        :::::::::::::::::::::::::::::::::::::::::::::::::::::::
        CACCCAAAAGTGCTCAGACCTTGTCTAGTTATTTATAAACTGTATGTACCTCCCTC(A)60

            2090          .          .          .          .       2140
        CTCTCCTATCACTGCTTTCCTACTCTCCTTTTATCTCACTCTAGTCCAGGTGCCAAGAAT

            2150          .          .          .          .       2200
        TTCCCTTCTACCCTCTATTCTCTTGTGTCTGTAAGTTACAAAGTCAGGAAAAGTCTTGGC

            2210          .          .          .          .       2260
        TGGACCCCTTTCCTGCTGGGTGGATGCAGTGGTCCAGGACTGGGGTCTGGCCCAGGTTT

            2270          .          .          .          .       2320
        GAGGGAGAAGGTTGCAGAGCACTTCCCACCTCTCTGAATAGTGTGTATGTGTTGGTTTAT

            2330          .
        TGATTCTGTAAATAAGTAAAATGACAA
```

**Figure 5** Nucleotide sequence of the 3′ end of the c-*fgr* cDNA clone pFa1, with predicted amino-acid sequence. The nucleotide sequence of the 3′ end of the c-*fgr* cDNA clone pFc11 is also shown. Putative polyadenylation signals are underlined.

**Figure 6** Northern blot of polyadenylated RNA isolated from Raji (lane a) and BL2–B95/1 (lane b) cells, hybridised with $^{32}$P-labelled antisense RNA synthesised from Bam HI-linearised pF3.4 DNA. Sizes of transcripts are indicated in kilobases.

recently-established EBV-negative BL cell lines with the B95.8 strain of EBV results in a dramatic elevation of levels of two c-*fgr* transcripts, of 2.9 kb and 3.5 kb. A similar pattern of transcripts has been described in the human B-lymphocyte cell line IM-9, derived by *in vitro* infection of normal peripheral blood B-lymphocytes with EBV (Inoue *et al.*, 1987). Cheah *et al.* (1986) were unable to find c-*fgr* transcripts in the EBV-negative BL cell lines which they studied, but we find that low levels are detectable in the EBV-negative parent cell lines BL2, BL31 and BL41. Many of the changes in cellular phenotype which accompany EBV-conversion resemble changes seen during normal B-lymphocyte activation, most strikingly the acquisition of cell surface markers such as CD23 and Blast-1 (Swendeman & Thorley-Lawson, 1987). These results raise the possibility, therefore, that the c-*fgr* protein is involved in the pathway of normal B-lymphocyte activation, perhaps as part of a protein phosphorylation cascade which transduces signals leading to B-lymphocyte differentiation and proliferation. Transcripts of the c-*fgr* gene are not limited to B-lymphocytes, though, having also been detected in well-differentiated monocytic cells (Willman *et al.*, 1987) in lung (Tronick *et al.*, 1985) and in placenta (Nishizawa *et al.*, 1986). It is not known which cell type(s) express c-*fgr* in the latter two tissues, but expression may be a reflection of their haematopoietic component.

The c-*fgr* gene is a member of a family which also includes c-*src* (Tanaka *et al.*, 1987), *fyn* (Semba *et al.*, 1987), *hck* (Quintrell *et al.*, 1987), *lck* (Voronova & Sefton, 1986), *lyn* (Yamanashi *et al.*, 1987), c-*tkl* (Strebhardt *et al.*, 1987) and c-*yes* (Sukegawa *et al.*, 1987). Unlike other tyrosine kinases such as c-*erb-B* and c-*fms* these proteins do not appear to be cell surface receptors, since they are thought to be located at the cytoplasmic surface of the plasma membrane (Pellman *et al.*, 1985). The c-*fgr* gene resembles *fyn*, *hck*, *lck*, *lyn* and c-*tkl* in having a restricted expression pattern, in contrast to c-*src* and c-*yes* whose expression is more widespread.

The proteins encoded by this gene family all have a highly conserved carboxyl-terminal domain which encodes tyrosine kinase activity by analogy with pp60$^{c\text{-}src}$. The amino-terminal domains are quite diverged, however, and nucleotide sequence data presented here shows that the amino-terminal domain of the c-*fgr* protein is also quite different from those of the other members of the family. This sequence divergence may indicate that the various family members, expressed in different cell types, have different substrate specificities. It is unlikely that it engenders differences in intracellular location since all members of the family, including the predicted c-*fgr* protein, as shown here, retain a glycine residue at position two. In pp60$^{c\text{-}src}$ this residue is the target for post-translational myristylation, which is necessary for the localisation of the protein to the inner surface of cytoplasmic membranes (Pellman *et al.*, 1985). It is likely, then, that the c-*fgr* protein shares the same intracellular location as pp60$^{c\text{-}src}$. It is also noteworthy that against the general background of diversity in amino-terminal sequences, the predicted *lyn*, *hck*, and *fyn* proteins (but not c-*src* or c-*yes*) contain peptides with homology to the sequence Tyr-Gly-Pro-Asp-Pro-Thr-Lys found at positions 34–40 in the predicted c-*fgr* protein (Figure 4). In addition, the *fyn* protein has homology to the sequence Ile-Pro-Asn-Tyr-Ser-Asn-Phe found at positions 50–57 in the c-*fgr* protein. The functional significance of these peptides, if any, is unclear.

Inoue *et al.* (1987) and Katamine *et al.* (1988) have recently reported nucleotide sequence derived from c-*fgr* cDNA clones. The nucleotide sequence of the 5′ end of the c-*fgr* cDNA clone pFd97 (Figures 2 and 3), is identical to that reported by Inoue *et al.* (1987), starting 3 bp downstream of their sequence. However, both our sequence and that of Inoue *et al.* (1987) differ in the 5′ untranslated region from that reported by Katamine *et al.* (1988). This latter sequence was derived from a cDNA clone representing an incompletely processed c-*fgr* transcript containing intron 2. It is therefore likely that the sequence of the 5′ untranslated region reported by Katamine *et al.* (1988) is in fact derived from intron 1, and that the sequence shown in Figure 3 is that of the 5′ untranslated region of the mature mRNA.

The 5′ untranslated regions of the mRNAs encoding different members of the tyrosine kinase family may have a role in regulating their differential expression. Marth *et al.* (1988) have recently shown that the 5′ untranslated region of *lck* mRNA contains AUG codons which reduce the efficiency of translation from the authentic initiation codon. As shown here, the 5′ untranslated region of the c-*fgr* mRNA contains two AUG codons, both of which are out-of-frame with respect to the initiating AUG. One of these, at nucleotides 101–103, obeys the rules of Kozak (1987) and so could be recognised by the 40S mammalian ribosomal subunit and be used to initiate translation, masking the authentic AUG. Kozak (1987) has noted that 65% of sequenced proto-oncogene mRNAs have AUGs in their 5′ untranslated regions, in contrast to fewer than 10% of 700 other mammalian genes surveyed, suggesting that cells might regulate the use of translational start sites in these mRNAs.

We have also demonstrated, by the nucleotide sequencing of c-*fgr* cDNA clones, that the mature c-*fgr* mRNA can be polyadenylated at either of two sites. The upstream polyadenylation signal, at nucleotides 2054–2059 does not encode the usual AAUAAA consensus sequence (Proudfoot & Brownlee, 1976), but UAUAAA, a sequence which has also been found to be used as a polyadenylation signal in the gene encoding hepatitis B virus surface antigen (McLauchlan *et al.*, 1985). The c-*fgr* cDNA clones of Inoue *et al.* (1987) and Katamine *et al.* (1988) have the extended 3′ end of pFa1, and the data of Katamine *et al.* (1988) show that the RNA species from which their cDNA clones derive are polyadenylated 13 bp downstream of the 3′ end of pFa1, presumably using the non-consensus polyadenylation signal AGUAAA encoded at nucleotides 2335–2340. This sequence is also used as a polyadenylation signal in the genomes of baboon erythroblastosis virus and mouse mammary tumour virus (McLauchlan *et al.*, 1985). The sequence AAUAAG encoded at nucleotides 2331–2336 is probably non-functional, since it has been shown not to direct polyadenylation of transcripts of a mutant α2-globin gene in a case of α-thalassaemia (Higgs *et al.*, 1983). On the basis of the numbers of cDNA clones of each type isolated, the downstream polyadenylation site appears to be used most frequently, Alternative polyadenylation does not account for the size difference between the 2.9 kb and 3.5 kb c-*fgr* transcripts but probably contributes to heterogeneity in the lower molecular weight band on Northern blots. This result agrees with recent data from Katamine *et al.* (1988), which suggests that the 3.5 kb transcript is an incompletely processed precursor, containing intron 2 of the c-*fgr* gene. Several genes have been characterised which contain alternative polyadenylation sites. In some cases, such as the bovine and human kininogen genes (Kitamura *et al.*, 1985) and the immunoglobulin μ heavy chain gene (Rogers *et al.*, 1980), the use of alternative polyadenylation sites generates proteins with different carboxyl-termini. In the case of the immunoglobulin μ heavy chain gene, polyadenylation site selection is developmentally regulated. The c-*fgr* gene resembles the rat disulphide isomerase gene (Edman *et al.*, 1985) and the hamster HMG CoA reductase gene (Reynolds *et al.*, 1984), however, in which the choice of polyadenylation site does not affect the protein product. It is not clear whether the choice of polyadenylation sites in the case of c-*fgr* is regulated. If it were, it could influence the stability of c-*fgr* mRNA in different cell types and thus be a mechanism for regulation of c-*fgr* gene expression.

Finally, the availability to us of cDNA clones representing 5′ sequences of the c-*fgr* mRNA now allows us to map the limits of the c-*fgr* transcription unit in cosmid genomic clones (our unpublished data), in order to study the basis of the regulation of c-*fgr* gene expression during EBV-conversion, and B-lymphocyte activation. In addition, the predicted amino-acid sequence of divergent portions of the c-*fgr* protein allows the synthesis of peptides for production of specific antisera with which to study the expression and function of this protein during the activation of B-lymphocytes.

This work was funded by the Cancer Research Campaign. We thank Professor R.K. Craig for helpful discussions and critical reading of this manuscript.

### References

BENTON, W.D. & DAVIS, R.W. (1977). Screening λgt recombinant clones by hybridisation to single plaques *in situ*. *Science*, **196**, 180.

CALENDER, A., BILLAUD, M., AUBRY, J.P., BANCHEREAU, J., VUILLAME, M. & LENOIR, G.M. (1987). Epstein–Barr virus (EBV) induces expression of B-cell activation markers on *in vitro* infection of EBV-negative B-lymphoma cells. *Proc. Natl Acad. Sci. USA*, **84**, 8060.

CHEAH, M.S.C., LEY, T.J., TRONICK, S.R. & ROBBINS, K.C. (1986). *fgr* proto-oncogene mRNA induced in B-lymphocytes by Epstein–Barr virus infection. *Nature*, **319**, 238.

CHIRGWIN, J.M., PRZYBYLA, A.G., MACDONALD, R.J. & RUTTER, W.J. (1979). Isolation of biologically active ribonucleic acid from sources enriched in ribonuclease. *Biochemistry*, **18**, 5294.

CRAIG, R.K., BROWN, P.A., HARRISON, O.S., MCILREAVY, D. & CAMPBELL, P.N. (1976). Guinea pig milk protein synthesis: isolation and characterisation of mRNAs from lactating mammary gland and identification of caseins and pre-α-lactalbumin as translation products in heterologous cell-free systems. *Biochem. J.*, **160**, 57.

EDMAN, J.C., ELLIS, L., BLACHER, R.W., ROTH, R.A. & RUTTER, W.J. (1985). Sequence of protein disulphide isomerase and implications of its relationship to thioredoxin. *Nature*, **317**, 267.

FEINBERG, A.P. & VOGELSTEIN, B. (1984). A technique for radiolabelling DNA restriction endonuclease fragments to high specific activity. *Anal. Biochem.*, **137**, 266.

HIGGS, D.R., GOODBOURN, S.E.Y., LAMB, J., CLEGG, J.B., WETHERALL, D.J. & PROUDFOOT, N.J. (1983). α-Thalassaemia caused by a polyadenylation signal mutation. *Nature*, **306**, 398.

HUMPHRIES, S., WHITTALL, R., MINTY, A., BUCKINGHAM, M. & WILLIAMSON, R. (1981). There are approximately 20 actin genes in the human genome. *Nucl. Acids Res.*, **9**, 4895.

HUYNH, T.V., YOUNG, R.A. & DAVIS, R.W. (1985). Constructing and screening cDNA libraries in λgt 10 and λgt11. In *DNA Cloning: A Practical Approach, Vol. 1*, Glover, D.M. (ed) p. 49. IRL Press: Oxford.

INOUE, K., IKAWA, S., SEMBA, K., SUKEGAWA, J., YAMAMOTO, T. & TOYOSHIMA, K. (1987). Isolation and sequencing of cDNA clones homologous to the v-*fgr* oncogene from a human B lymphocyte cell line, IM-9. *Oncogene*, **1**, 301.

KATAMINE, S., NOTARIO, V., RAO, C.D. & 4 others (1988). Primary structure of the human *fgr* proto-oncogene product $p55^{c\text{-}fgr}$. *Mol. Cell. Biol.*, **8**, 259.

KITAMURA, N., KITAGAWA, H., FUKUSHIMA, D., TAKAGAKI, Y., MIYATA, T. & NAKANISHI, S. (1985). Structural organisation of the human kininogen gene and a model for its evolution. *J. Biol. Chem.*, **260**, 8610.

KOZAK, M. (1987). An analysis of 5′-noncoding sequences from 699 vertebrate messenger RNAs. *Nucl. Acids Res.*, **15**, 8125.

MARTH, J.D., OVERELL, R.W., MEIER, K.E., KREBS, E.G. & PERLMUTTER, R.M. (1988). Translational activation of the *lck* proto-oncogene. *Nature*, **332**, 171.

MCLAUCHLAN, J., GAFFNEY, D., WHITTON, J.L. & CLEMENTS, J.B. (1985). The consensus sequence YGTGTTYY located downstream from the AATAAA signal is required for efficient formation of mRNA 3′ termini. *Nucl. Acids Res.*, **13**, 1347.

MURRAY, R.J., YOUNG, L.S., CALENDER, A. & 4 others (1988). Different patterns of Epstein–Barr virus gene expression and of cytotoxic T-cell recognition in B-cell lines infected with transforming (B95.8) or non-transforming (P3HR1) virus strains. *J. Virol.*, **62** (in press).

NISHIZAWA, M., SEMBA, K., YOSHIDA, M.C., YAMAMOTO, T., SASAKI, M. & TOYOSHIMA, K. (1986). Structure, expression and chromosomal location of the human c-*fgr* gene. *Mol. Cell. Biol.*, **6**, 511.

PARKER, R.C., MARDON, G., LEBO, R.V., VARMUS, H.E. & BISHOP, J.M. (1985). Isolation of duplicated human c-*src* genes located on chromosomes 1 and 20. *Mol. Cell. Biol.*, **5**, 831.

PELLMAN, D., GARBER, E.A., CROSS, F.R. & HANAFUSA, H. (1985). An N-terminal peptide from $p60^{src}$ can direct myristylation and plasma membrane localization when fused to heterologous proteins. *Nature*, **314**, 374.

PROUDFOOT, N.J. & BROWNLEE, G.G. (1976). 3′ non-coding region sequences in eukaryotic messenger RNA. *Nature*, **263**, 211.

QUEEN, C. & KORN, L.J. (1984). A comprehensive sequence analysis program for the IBM personal computer. *Nucl. Acids Res.*, **12**, 581.

QUINTRELL, N., LEBO, R., VARMUS, H. & 5 others (1987). Identification of a human gene (*HCK*) that encodes a protein-tyrosine kinase and is expressed in hemopoietic cells. *Mol. Cell. Biol.*, **7**, 2267.

RANSOM, J.T., HARRIS, L.K. & CAMBIER, J.C. (1986). Anti-Ig induces release of inositol 1,4,5-trisphosphate, which mediates mobilization of intracellular $Ca^{++}$ stores in B lymphocytes. *J. Immunol.*, **137**, 708.

REYNOLDS, G.A., BASU, S.K., OSBORNE, T.F. & 5 others (1984). HMG CoA reductase: a negatively regulated gene with unusual promoter and 5′ untranslated regions. *Cell*, **38**, 275.

ROGERS, J., EARLY, P., CARTER, C. & 4 others (1980). Two mRNAs with different 3′ ends encode membrane bound and secreted forms of immunoglobulin $\mu$ chain. *Cell*, **20**, 303.

ROWE, M., ROONEY, C.M., EDWARDS, C.F., LENOIR, G.M. & RICKINSON, A.B. (1986). Epstein–Barr virus status and tumour cell phenotype in sporadic Burkitt's lymphoma. *Int. J. Cancer*, **37**, 367.

SEMBA, K., NISHIZAWA, M., MIYAJIMA, N. & 6 others (1986). *yes*-related proto-oncogene, *syn*, belongs to the protein-tyrosine kinase family. *Proc. Natl Acad. Sci. USA*, **83**, 5459.

STREBHARDT, K., MULLINS, J.I., BRUCK, C. & RÜBSAMEN-WAIGMANN, H. (1987). Additional member of the protein-tyrosine kinase family: The *src*- and *lck*-related protooncogene c-*tkl*. *Proc. Natl Acad. Sci. USA*, **84**, 8778.

SUKEGAWA, J., SEMBA, K., YAMANASHI, Y. & 4 others (1987). Characterisation of cDNA clones for the human c-*yes* gene. *Mol. Cell. Biol.*, **7**, 41.

SWENDEMAN, S. & THORLEY-LAWSON, D.A. (1987). The activation antigen BLAST-2, when shed, is an autocrine BCGF for normal and transformed B cells. *EMBO. J.*, **6**, 1637.

TAKEYA, T. & HANAFUSA, H. (1983). Structure and sequence of the cellular gene homologous to the RSV *src* gene and the mechanism for generating the transforming virus. *Cell*, **32**, 881.

TAYLOR, J.B., CRAIG, R.K., BEALE, D. & KETTERER, B. (1984). Construction and characterisation of a plasmid containing complementary DNA to mRNA encoding the N-terminal amino acid sequence of the rat glutathione transferase Ya subunit. *Biochem. J.*, **219**, 223.

TANAKA, A., GIBBS, C.P., ARTHUR, R.R., ANDERSON, S.K., KUNG, H-J. & FUJITA, D.J. (1987). DNA sequence encoding the amino-terminal region of the human c-*src* protein: implications of sequence divergence among *src*-type kinase oncogenes. *Mol. Cell. Biol.*, **7**, 1978.

THORLEY-LAWSON, D.A. & MANN, K.P. (1985). Early events in Epstein–Barr virus infection provide a model for B-cell activation. *J. Exp. Med.*, **162**, 45.

TRONICK, S.R., POPESCU, N.-C., CHEAH, M.S.C. & 5 others (1985). Isolation and chromosomal localization of the human *fgr* proto-oncogene, a distinct member of the tyrosine kinase gene family. *Proc. Natl Acad. Sci. USA*, **82**, 6595.

VORONOVA, A.F. & SEFTON, B.M. (1986). Expression of a new tyrosine protein kinase is stimulated by retrovirus promoter insertion. *Nature*, **319**, 682.

WATSON, C.J. & JACKSON, J.F. (1985). An alternative procedure for the synthesis of double-stranded cDNA for cloning in phage and plasmid vectors. In *DNA Cloning: A Practical Approach*, *Vol. I*. Glover, D.M. (ed) p. 79. IRL Press: Oxford.

WILLMAN, C.L., STEWART, C.C., GRIFFITH, J.K., STEWART, S.J. & TOMASI, T.B. (1987). Differential expression and regulation of the c-*src* and c-*fgr* protooncogenes in myelomonocytic cells. *Proc. Natl Acad. Sci. USA*, **84**, 4480.

YAMANASHI, Y., FUKUSHIGE, S-H., SEMBA, K. & 5 others (1987). The *yes*-related cellular gene *lyn* encodes a possible tyrosine kinase similar to $p56^{lck}$. *Mol. Cell. Biol.*, **7**, 237.

ZEUTHEN, J. (1983). Epstein–Barr virus transformation: biological and functional aspects. *Adv. Viral. Oncol.*, **3**, 183.